# The Construct Validity of the Childhood Joint Attention Rating Scale (C-JARS) in School-Aged Autistic Children

**DOI:** 10.1007/s10803-023-06051-1

**Published:** 2023-07-22

**Authors:** Sandy L. Birkeneder, Jennifer Bullen, Nancy McIntyre, Matthew C. Zajic, Lindsay Lerro, Marjorie Solomon, Nicole Sparapani, Peter Mundy

**Affiliations:** 1 School of Education and the MIND Institute, University of California, Davis, One Shields Ave., Davis, CA 95616 USA; 2 MIND Institute and Department of Psychiatry and Behavioral Science, University of California, Davis, 2825 50th Street, Sacramento, CA 95817 USA; 3 Communication Sciences and Disorder, University of Central Florida, 12805 Pegasus Drive, Orlando, FL USA; 4 Teachers College, Health and Behavior Studies, Columbia University, 525 W. 120th Street, Box 223, New York, NY 10027 USA; 5 The Swain Center, Santa Rosa, 795 Farmers Lane, Suite 23, Santa Rosa, CA 95405 USA

**Keywords:** Autism spectrum disorder, Diagnostics, Parent-report measure, Symptoms, Joint attention, Prosocial behaviors

## Abstract

Preliminary evidence from the Childhood Joint Attention Rating Scale (C-JARS; Mundy et al., 2017) suggests symptoms related to diminished joint attention and the spontaneous sharing of experience with others can be assessed with a parent-report measure in children and adolescents with autism. This study was designed to expand on the previous study by examining the validity of both a Social Symptom (SS) and a Prosocial (PS) scale of the C-JARS in a study of school-aged autistic children (*n*  = 89) with and without co-occurring intellectual disability (ID), as well as an age matched neurotypical sample (*n*  = 62). Results indicated that both C-JARS scales were sensitive and specific with respect to identifying the diagnostic status of the children. In addition, the PS scale was sensitive to differences in cognitive abilities (IQ) and sex differences in the autism group. These results are consistent with the hypothesis that joint attention and spontaneous sharing of experience symptoms are not only characteristic of preschool children with autism but may also constitute a developmentally continuous dimension of the social phenotype of autism that can be measured in school-aged children.

## Introduction

Autism is a neurodevelopmental condition that develops in the first years of life and affects as many as one in forty-four school-aged children (Maenner et al., 2021). Some of the earliest observable symptoms of autism involve differences in joint attention (Franchini et al., 2019; Gotham et al., 2007; Mundy & Bullen, 2022). Joint attention is the ability to socially coordinate attention with another person in relation to a third object or event (Mundy, 2018). Differences in the development of joint attention begin to be measurable in the infant siblings of children with autism between 6 and 12-months (see Mundy & Bullen, 2022 for a review). Differences in joint attention development between 6 and 18-months also begin to distinguish toddlers with an increased likelihood of receiving a diagnosis of autism from peers who receive a diagnosis for other early onset communication disorders (Franchini et al., 2019; Nystrom, 2019; Stallworthy, 2022).

Joint attention development reflects cognitive and motivation processes (Mundy, 1995; Mundy & Newell, 2007). Cognitive processes involve the perceptual and mental capacity to adopt a common frame of reference with other people (Mundy, 2018; O’Madagain & Tomasello, 2021; Siposova & Carpenter, 2019), and motivation processes involve the prosocial and affiliative tendency to spontaneously share experience with others (Mundy et al., 1992; Van Hecke et al., 2007; Venezia et al., 2004). Indeed, joint attention is associated with parent’s ratings of their children’s relatedness to others (Mundy et al., 1994) and symptoms are associated with an attenuated tendency of autistic children to share positive affective experiences with others (Kasari et al., 1990). To some extent the fifth edition of the Diagnostic and Statistical Manual of Mental Disorders (DSM-V) describes the attenuated tendency of autistic children to share experience with others as a symptom in its description of “Persistent deficits in social communication and social interaction across multiple contexts” in item A.1, which is “Deficits in social-emotional reciprocity, ranging, for example from back and forth conversation, to reduced sharing of interests, emotions or affect; to failure to initiate or respond to social interactions” (p. 50, American Psychiatric Association, 2013)*.*

The *Autism Diagnostic Observation Schedule-Revised* (ADOS-2; Lord et al., 2012) more explicitly include joint attention in its operational definition of the social symptoms on the Social Affect (SA) scale of modules 1 and 2 used with young children. The SA scale in these modules include response to joint attention, initiation of joint attention, showing, pointing, unusual eye contact, and gesture items that display evidence of converging on a joint attention factor within this ADOS-2 scale (Gotham et al., 2007, 2008). Module 3 of the ADOS-2 also includes items such as unusual eye contact, facial expressions directed to examiner, and shared enjoyment interaction (Lord et al., 2012), which may be expected to relate to the social-communicative function of joint attention (Mundy et al., 2017).

Although symptoms of atypical joint attention and spontaneous sharing of experience are observable in infancy and preschool children, it has not been clear if these symptoms are apparent in school-aged autistic children (Lord & Jones, 2012). Recent studies, however, have begun to report data on the development of joint attention measures for research with older children. For example, experimental measures of information processing and cortical activation patterns during joint attention have been developed (Mundy et al., 2016; Oberwelland et al., 2017), as has a classroom observation coding system which assesses joint attention as an index of student–teacher engagement (Dykstra Steinbrenner & Watson, 2015). Moreover, Nowell and colleagues (2018) published psychometric data on a 16-item measure called the *Joint Attention Protocol (JA-Protocol)*, which is a live-coded observation measure of joint attention behavioral development for two to twelve-year-old children. The IJA and RJA measures of the JA-Protocol were significantly correlated with scores for comparable ADOS SA scale items in a sample of autistic children. Bean and Eigsti (2012) have also developed an observation measure of responding to joint attention for children and adolescents.

The data from these measures suggest that the construct of joint attention can be usefully measured after the preschool period. However, all of these measures require direct observations or laboratory paradigms for their implementation. These can provide valuable information. However, the considerable time and effort of their administration and scoring can limit their use in large scale, multidisciplinary studies. Instead, parent report measures such as the Social Responsiveness Scale (Constantino & Gruber, 2012), or the Social Communication Questionnaire (Lord & Rutter, 2003) are more efficient and are often used to assess the social outcomes of school-aged children with autism (e.g., Charman et al., 2017; Dinstein et al., 2020). The available parent report assessments, though, tend to be broad-band measures that are not designed to assess specific symptom dimensions. Alternatively, narrow-band, parent report measures of theoretically specifiable symptom dimensions of autism, such as developmental differences in joint attention, may be expected to compliment broadband measures. For example, such a narrow-band assessment may facilitate the needed study of the continuity of a specific social symptom dimension across preschool and school-aged development in autistic children (Lord & Jones, 2012; Mundy, 2018), or as a theoretically meaningful outcome measure of early intervention that target joint attention related behaviors (e.g., Kasari et al., 2008; Rollins et al., 2021).

The Childhood Joint Attention Rating Scale (C-JARS, Mundy et al., 2017) was developed to address the need for such a parent report measure. The C-JARS also addresses the need to consider verbal behavior in measuring joint attention in children. Existing childhood measures focus solely on the assessment of non-verbal measures of joint attention (Bean & Eigsti, 2012; Nowell et al., 2018). However, joint attention is not simply a behavior it is a mental process (Mundy & Newell, 2007) and, with development, shared attention and sharing experience is also likely indicated through verbalizations (Mundy et al., 2017). Hence, the C-JARS was designed to assess both nonverbal and verbal behaviors. Several studies also indicate that joint attention is related to the development of prosocial behavior in typical development (Stout et al., 2021; Vaughan Van Hecke et al., 2007) and in autism (Travis et al., 2001). Therefore, the development of joint attention and spontaneous sharing of experience in autistic children may best be measured in terms of a scale of continued problems with joint attention (negative social symptoms) or an increase in the positive or prosocial use of joint attention and sharing experience. Therefore, the C-JARS was designed to provide both a symptom and prosocial index of joint attention and social sharing of experience in children. The latter also contributes to a need to address the surfeit of prosocial outcome measures available for autism research (Yager & Iarocci, 2013).

A preliminary study of the initial 60-item pool used to develop the C-JARS was conducted to determine which, if any of the items would converge on a single factor (construct) that accounted for a significant portion of the variance in parent reports for 52 eight- to sixteen-year-old autistic children and 34 age-matched non-autistic comparison children (Mundy et al., 2017). The results indicated that a large subset of 56 of the 60 items converged on a single factor, explaining 48.7% of the item covariance. A single factor comprised of these 56 items accounted for 40% in the autism sample and 48% in the comparison sample. Moreover, a factor-based scale score displayed a sensitivity of 88% and specificity of 62% with respect to a control sample that included eleven children with symptoms of ADHD. These data provided a first step in the development and evaluation of the C-JARS.

The preliminary study (Mundy et al., 2017), however, did not examine the psychometric characteristics of the separate Social Symptom scale (SS) and Prosocial (PS) scale scores of the C-JARS. Moreover, that study only included children that had IQ test performance greater than 75. However, approximately 33% of the population of school-aged autistic children have a co-occurring intellectual disability (Maenner et al., 2021). So, this study was designed to further the examination of the psychometric characteristics and construct validity of the C-JARS by examining data from the SS and PS scales in a second, larger sample of autistic children with and without co-occurring intellectual disability, as well as in a neurotypical (NT) comparison sample.

The specific aim of this study was to test several hypotheses pertinent to an examination of the psychometric characteristics of the C-JARS and its construct validity. Based on previous data (Mundy et al., 2017) we expected that analyses would indicate that a single factor accounts for a substantial portion of the variance in parent report on the C-JARS and that the SS and PS scales would exhibit acceptable levels of reliability. In terms of construct validity, the first hypothesis was that the C-JARS scores would reflect a characteristic of childhood social development that would significantly distinguish autistic children from their neurotypical peers (Mundy et al., 2017). Also based on previous findings (Mundy et al., 2017), the second hypothesis was that the C-JARS scores would demonstrate convergent and divergent criterion related validity with other clinical and parent report measures of social symptoms. In this regard several assumptions were tested.

The construct of joint attention and sharing experience with other people is theoretically and empirically associated with a sense of affiliation, social bonding, and relatedness between social partners (e.g., Bothby et al., 2014; Gable et al., 2004; Mundy et al., 1994; Phillips et al., 2019; Wolf et al., 2016). Therefore, we expected parent report on the C-JARS PS scale would be significantly associated with parent reports on the Affiliation scale of the Temperament in Middle Childhood Questionnaire (TMCQ; Simonds & Rothbart, 2004), which measures confiding behaviors, evidence of liking others and enjoying interacting with friends, but does not include items specific to joint attention.

Based on previous research (Mundy et al., 2017) we also expected that parent report on the C-JARS would be associated with independent clinical observations of social symptoms from the ADOS-2. More specifically we expected that C-JARS SS scale scores would be positively correlated with ADOS-2 SA scores, and the PS scale scores would be negatively correlated with SA scores in autistic children. We also expected that the C-JARS would not be associated with non-social behaviors observations of ADOS-2 RRB scale.

The Vineland Adaptive Behavior Scales Socialization score (Vineland-II; Sparrow et al., 2005) is a recognized index of aspects of the social phenotype of autistic children (Bolte et al., 2002). Therefore, if the C-JARS also provides valid information about an important aspect of the social phenotype of autism in childhood we would expect that the C-JARS SS and PS scale scores would be negatively and positively associated with the Vineland-II Socialization scale scores, respectively.

Finally, joint attention and spontaneous sharing of experience is hypothesized to reflect a unique dimension in childhood development (Moll et al., 2021; Mundy & Bullen, 2022). However, this dimension may be expected to be associated with other factors such as intellectual development, language development, or anxiety (Bottema-Buetel, 2016; Lei & Ventola, 2018; Sanso et al., 2021). Nevertheless, in terms of the evidence of its construct validity we expected that the association of these factors with the C-JARS would not explain the predicted associations between the C-JARS and the ADOS-2, Vineland-II or TMCQ Affiliation scale scores in the sample of autistic children.

## Methods

### Participants

The sample was comprised of 151 school-aged children, which included 89 children diagnosed with autism and 62 age-matched neurotypical (NT) children (see Table 1) who were participating in the Time 4 assessment of a larger longitudinal study, the Autism Phenome Project (APP), at the UC Davis MIND Institute. The age range in the autism sample was 9 years and 4 months to 13 years and 9 months. The age range in the neurotypical sample was 10 years and 2 months to 13 years and 9 months. The ancestral demographic characteristics for the autism sample were, 71% White, 5% Black, 5% Asian, 17% Hispanic, 1% Native American, and 14% mixed or other. Commensurately, the ancestral diversity of the neurotypical sample were, 72% White, 0% Black, 4% Asian, 26% Hispanic, 0% Native American, and 20% mixed or other. The reported level of education for the mothers/fathers of the autistic children were, respectively, 6%/16% high school or less, 18%/16% some college, 24%/18% technical or associate degree, 38%/34% bachelor’s degree, 12%/10% master’s degree, and 3%/6% doctoral degree. The reported level of education for the mothers/fathers for the neurotypical children were 10%/22% high school or less, 8%/12% some college, 20%/14% technical or associate degree, 35%/33% bachelor’s, 20%/16% master’s degree, and 8%/4% doctoral degree.

**Table 1:** Group data for age, sex, autism symptoms, and cognitive status

Characteristics	Lower IQ Autism		Higher IQ Autism		NT	
	*n*	M (SD)	*n*	M (SD)	*n*	M (SD)
Age	36	11.59 (1.07)	53	11.52 (0.84)	62	11.47 (0.78)
Sex						
Female	7	19.4%	12	22.6%	22	35.5%
Male	29	80.6%	41	77.4%	40	64.5%
ADOS-2						
SA Total	35	14.11 (3.65)	51	9.29 (3.37)		
RRB Total	35	5.91 (1.65)^b^	51	3.31 (1.93)		
DAS-II						
FSIQ	36	57.83 (14.62)^b,c^	53	103.74 (15.50)^a,c^	62	113.73 (12.95)^a,b^
Verbal IQ	31	50.48 (17.65)^b,c^	53	101.85 (18.88)^a,c^	62	115.98 (11.04)^a,b^
Nonverbal IQ	35	64.77 (15.37)^b,c^	53	104.32 (14.16)^a^	62	110.19 (14.42)^a^

Lower IQ autism group, IQ < 80. Higher IQ autism, IQ > 79. *ADOS*-2 Autism Diagnostic Observation Schedule, Second Edition. *SA* Social Affect score on the ADOS-2. *RRB* Restricted and Repetitive Behavior scores on the ADOS-2. *DAS*-II Differential Abilities Scales, Second Edition. *Autism* autism spectrum disorder. *NT* neurotypical

^a^Significantly different from Lower IQ autism group, *p* < .001

^b^Significantly different from Higher IQ autism group, *p* < .01

^c^Significantly different from NT group, *p* < .001

As previously noted this study was designed to examine the diagnostic reliability and validity of the C-JARS in groups of autistic children with and without co-occurring intellectual disability, as well as a NT comparison sample. A comparative categorical “group” design was used in this study, rather than examining IQ as a continuous variable. This was done to address the paucity of data reported in autism research that are specific to the group of children with co-occurring intellectual disabilities (Russell et al., 2019) and the consequent need to provide more explicit basic and clinical research information about these children (Lord et al., 2022). Thus, the autism group in this study was divided into Lower and Higher IQ subgroups. The Lower IQ autism group included 27 children with intellectual disability (IQ < 71) *and* 9 children with borderline intellectual disturbance (IQs 71–79). The Higher IQ autism group had 53 children with IQs > 79 (see Table 1).

### Procedures

Close contact was maintained with families throughout Time 1, 2, 3, 4, and 5 data collections for the APP, such that attrition rates did not exceed 25%. Inclusion criteria for autism in the APP were based on the NIH Collaborative Programs of Excellence in Autism standards. Participants had received a best estimate diagnosis of autism, PDD-NOS, or Asperger syndrome from a licensed site clinician. Participants then met the ADOS-2 cut-off score for autism or met the Autism Diagnostic Interview-revised (ADI-R; Lord, Rutter, & Le Couteur, 1994) criteria for autism on either the Social or Communication subscale, while being within two points of this criterion on the other subscale. The ADOS-2 was administered by a trained clinician and provided age-standardized calibrated severity scores and Total scores for social-communication symptoms on the SA scale and limited interests, repetitive behaviors, and sensory sensitivity on the Restrictive & Repetitive Behavior (RRB) scale. Participants needed to live with at least one biological parent, be English speaking and ambulatory, and have no severe motor, vision hearing or chronic health problems that would preclude them from being assessed. All parents participated in an institutional review board (IRB) approved informed consent process prior to participation in each phase of the longitudinal assessments. IRB review and oversite occurred throughout the course the study and all data were collected in accord with approved human subjects research protocols.

During the Time 4 assessment children and their families participated in multiple visits to a pediatric research clinic of a major urban medical center. During these visits, children in the autism group received medical exams and standardized diagnostic assessments. All children and parents were asked to complete a variety of behavioral questionnaires and children participated in cognitive, executive function and language assessments administered by trained clinicians, as well as fMRI protocols and the provision of blood specimens.

## Measures

### Childhood Joint Attention Rating Scale (C-JARS)

The C-JARS measures three types of behaviors associated with joint attention and the spontaneous sharing of experience (see Table 2 for examples). The first measure is *nonverbal gaze following and gaze directing behaviors*, which are central to preschool children and childhood measures of joint attention (e.g., Mundy et al., 2007; Nowell et al., 2018). The second measure is *children’s spontaneous verbalizations to confide or share their positive experiences with others*, which has a positive impact on the sense of relatedness between social partners (Boothby et al., 2014; Gable et al., 2004). The third measure is *children’s tendency to engage in joint action, collaborative and cooperative activities*, which are included because joint attention and joint action share common mental and social affiliative mechanisms (Metcalf & Terrace, 2013; Milward & Carpenter, 2018; Sebanz et al., 2009; Wolf et al., 2016).

**Table 2:** Examples from the social symptom (SS) and prosocial (PS) scales

Example items	Never	Seldom	Sometimes	Often	Always
Social symptom (SS) items					
14. S/he does not look up to acknowledge other people when interested in an object or event	0	1	2	3	4
21. S/he talks to others *without* looking at them	0	1	2	3	4
51. S/he has difficulty maintaining a back-and-forth conversation with others	0	1	2	3	4
Prosocial (PS) Items					
4. S/he directs your attention to share an experience about specific objects or events (such as food; an event in a book, a movie, or on the web; sometimes he/she hears, feels, sees, or smells, or tastes)	0	1	2	3	4
22. S/he shares exciting events with you that happened at school	0	1	2	3	4
27. S/he works cooperatively in groups of more than one other child to achieve a common goal	0	1	2	3	4

SS higher score indicates more consistent parent report of infrequent or atypical joint attention behaviors. PS higher score reflects the degree to which parents frequently observe joint attention and spontaneous sharing of experience in children

The initial study (Mundy et al., 2017) resulted in 56 items, but a single item with the lowest factor loading from that study was subsequently removed. This resulted in a 55-item measure used in this study.

All C-JARS items are scored on a 5-point rating scale (0 = Never, 1 = Seldom, 2 = Sometimes, 3 = Often and 4 = Always). The Social Symptom (SS) scale score is derived from 14 items and the Prosocial (PS) scale score is based on 41 items. The total item score for each scale is divided by the number of items to provide average SS and PS scores on a comparable scale. Higher average SS scores represent parents’ observations of more atypical and less frequent joint attention/sharing experience behaviors (see Table 2). Higher PS scores represent parent observations of more frequent child joint attention/sharing experience behaviors (see Table 2). To compute a total score the average SS scale score is subtracted from the average PS scale score for each child. This yields a Summary Joint Attention Scale (SJAS) score where negative scores are indicative of higher average symptom items scores than prosocial scores and positive scores are indicative of higher average prosocial item scores than symptom scores.

### Multidimensional Anxiety Scale for Children, Second Edition (MASC-2)

The parent-report version of the MASC-2 (March, 2013) assesses anxiety symptoms for 8- to 19-year-old children and adolescents. The measure consists of 39 items. Twelve items measure a Physical Symptom factor that includes symptoms of tension and restlessness (e.g., shaking, jumping, feeling weird) and somatic symptoms (e.g., chest tightness, sweating, dizziness). Nine items measure Social Anxiety including reports of fears about being called on in class, public performance, what other’s think, “looking stupid,” and being embarrassed. Nine items also Measure Harm avoidance with items about fear about acting without permission, vigilance about safety, and avoidance of upsetting circumstances. Finally, the MASC-2 also includes a 9-items scale for Separation Anxiety that includes fears about going to camps, being alone, especially at night, and separation from parents. The MASC-2 provides standardized T-scores for these four scales, as well as a Generalized Anxiety Disorder (GAD) Index, which differentiates children with GAD from the general population. Previous research indicates that the MASC-2 provides a reliable and valid index of anxiety on autistic school-aged children (Burrows et al., 2018; Schiltz et al., 2017).

### Temperament in Middle Childhood Questionnaire (TMCQ)

The TMCQ (Simonds & Rothbart, 2004; Simonds et al., 2007) is a 65-item, paper–pencil parent-report measure initially designed for 7–10-year-old children, but with psychometric characteristics indicating it is applicable to children up to age 14 in child clinical samples (Kozlowski et al., 2022). It provides scores on four factor-based scaled scores (Social Affiliation, Surgency/Extraversion, Effortful Control, and Negative Affectivity) based on Rothbart’s (2001) model of temperament. The Social Affiliation scale was of particular interest in this study. It includes five items measuring parent’s rating of children’s: (1) confiding in others, (2) liking being with others, (3) liking to feel close to others, (4) indicating friends are very important, and (5) interacting with friends every day. Surgency measures approach and activity tendency with items assessing: (1) activity level, (2) high intensity pleasure (e.g., rides a bike downhill very fast), (3) Positive approach anticipation (e.g., excitement related to future pleasurable activity), (4) smiling and laughter, (5) Assertiveness (e.g., speaks to gain or maintain attention in social situations), and (6) shyness (negatively scored). Effortful Control assesses: (1) Inhibitory control, (2) Attention Focusing (e.g., sustained attention), Activation Control (e.g., the ability to initiate action despite difficulties), (4) Openness to ideas and experience, and (5) Low Intensity Pleasure (e.g., pleasure in novelty, complexity, or low intensity stimuli). Negative Affectivity measures: (1) Anger/Frustration (e.g., in response to task interruption), (2) Sadness, (3) Soothability (e.g., recovery rate from distress or excitement), (4) Fear, and (5) Discomfort (e.g., negative affect related to sensory stimulation).

The TMCQ has demonstrated good internal consistency estimates for all measures used in this study (alpha > 0.70; Nystrom & Bengstsson, 2017), including evidence from research studies involving autistic children (Lee et al., 2020).

### Vineland Adaptive Behavior Scales, Second Edition (Vineland-II)

The Vineland-II (Sparrow et al., 2005) is a parent report measure of adaptive functioning skills with longstanding evidence of psychometric validity for autism research (Perry & Factor, 1989). The Vineland-II includes three subscales—Communication, Daily Living Skills, and Socialization Behaviors—that have often been used in studies examining the behavioral development of autistic children (Klin et al., 2007; Kraijer, 2000). Within the Communication subscale are individual scores for receptive and expressive language. This study will utilize the Socialization subscale scores, and the standardized “v” scores (Sparrow, 2011) for both receptive and language subscales (mean = 15, SD = 3) from the Communication subscale.

### Differential Abilities Scales, Second Edition (DAS-II)

The DAS-II (Elliott, 2007) provides a standardized measure of verbal and nonverbal cognitive functioning in children and adolescents. The test produces a Full-scale IQ (FSIQ) score and several subtests that comprise three cluster scores (Verbal Reasoning, Nonverbal Reasoning, and Spatial Ability). This study utilizes the FSIQ, Verbal and Nonverbal scores. The DAS-II demonstrates good reliability and is a valid measure of the cognitive status of autistic children (Kuriakose, 2014).

### Clinical Evaluation of Language Fundamentals, Fifth Edition (CELF-5)

The CELF-5 (Wiig et al., 2013) measures aspects of language development from 5 to 21 years of age. The test was administered by a trained assessor. In this study, the Receptive Language Index (RLI) and Expressive Language Index (ELI) composites of the CELF-5 were used as omnibus language measures. The RLI is a composite score derived from subscales assessing comprehension of Concepts and Directions, Word Classes, Sentence Structure and Semantics. The ELI is derived from subscales assessing Formulating Sentences, Word Structure, Sentence Assembly, and Recalling Sentences abilities. The CELF-5 was chosen because it has excellent psychometric properties and provides a standardized measure of language that is associated with individual differences in spontaneous speech in school-aged autistic children (Condouris et al., 2003; Coret & McCrimmon, 2015; Wiig et al., 2013).

### Data Analysis

The normality, homogeneity of variance, and multicollinearity of the C-JARS scores’ distributions within all groups were examined to ensure the data met criteria for parametric analyses. Next, psychometric analyses were conducted to re-examine the factor structure of the C-JARS in the data from this study. The reliability (internal consistency) of the C-JARS SS, PS, and SJAS scale scores were evaluated with Cronbach’s alpha (Cronbach, 1951). An analysis was conducted to examine the covariance among the C-JARS scale scores. Preliminary analyses were conducted to determine if variance associated with age or IQ needed to be considered in subsequent analyses.

To test the hypothesis that parent report on the C-JARS would distinguish autistic children from their neurotypical peers we analyzed the 2 C-JARS scores (SS & PS) with a 3 (Group) X 2 (Sex) multivariate analysis of variance (MANOVA). Planned pairwise group comparisons were conducted using Tukey’s HSD, and post-hoc comparisons for interactions used Bonferroni adjustments for multiple comparisons. Planned discriminant function analysis was used to examine the sensitivity and specificity of the C-JARS for identifying children in the autism group and NT comparison group. We also examined the receiver operator characteristics (ROC; Swets, 1996) associated with these analyses.

To examine the criterion related construct validity of the C-JARS Pearson correlation analyses were used to examine the prediction that C-JARS scores would be significantly associated with the TMCQ Affiliation scale scores, ADOS-2 SA Total scores, and the Vineland-II Socialization scale scores. Partial correlation analysis was also used to examine the prediction that C-JARS scale scores would not be associated with non-social RRB Total scores of the ADOS-2 and that correlations between the C-JARS scales scores and measures of language (e.g., CELF-5 scores) or anxiety (MASC-2 scores) would not explain the association between the C-JARS scales scores and TMCQ Affiliation, ADOS-2 SA, or Vineland-II Socialization scores. Finally, exploratory correlation analyses were conducted to examine the possible association of C-JARS scales scores with the following measures: TMCQ Surgency (extraversion), TMCQ Effortful Control, and TMCQ Negative Affect factor scores. Since the C-JARS was initially examined in a study of autistic children without intellectual disabilities (Mundy et al., 2017), a goal of this study was to begin to understand if the construct validity is equivalent for children with and without intellectual disability. To that end, the correlations pertinent to evaluating construct validity were computed separately for the two autism IQ subgroups. The hypotheses led to predictions of the directions of all the correlational tests of construct validity. Therefore, to control for the impact of the number of correlations calculated in this study on Type 1 error, the one-tailed alpha for correlations reported as significant in this study was set to 0.005 one-tailed, or a two two-tailed alpha at 0.01. By convention the two-tailed p-values are reported in this paper.

## Results

### Psychometric Analyses

A factor analysis with varimax rotation revealed a single factor solution accounted for 49.3% of the covariance in the C-JARS items in this study, which was similar to results previously reported for an independent sample (Mundy et al., 2017). No other factor accounted for more than 2.2% of the variance in this study.

Scale reliability analyses indicated that the internal consistency estimate (Cronbach’s alpha) for the SS scale in the full sample was 0.91, with subgroup-specific alphas being 0.75 in the Lower IQ autism group, 0.80 in the Higher IQ autism group, and 0.76 in the NT group. The internal consistency estimate for the PS scale in the full sample was 0.98, with subgroup-specific alphas being 0.95 in the Lower IQ autism group, 0.97 in the Higher IQ autism group, and 0.92 in the NT group. The SJAS is based on all items and best reflects the single factor of the C-JARS. Here, the alpha coefficients were 0.90, 0.93, 0.92 for the Lower and Higher IQ autism groups and the NT group, respectively.

The correlations between the SS and PS scale scores were examined to understand the degree to which they constituted distinct, overlapping, or redundant measures. As expected, these scale scores were negatively correlated and significant in the Lower IQ autism group (*r* = − 0.46, *p* < 0.001) and the Higher IQ autism group (*r* = − 0.68, *p* < 0.001). The correlation in the NT group was also negative (*r* = − 0.25, *p* = 0.05). The difference between these within group correlations was not significant, *p* = 0.09. The correlations of the SJAS scores with the SS scores in these three groups were uniformly negative (*rs* = − 0.82, − 0.89, and − 0.80, *ps* < 0.001) and uniformly positive with the PS scores, (*rs* = 0.87, 0.94, and 0.80, *ps* < 0.001) respectively, for the Lower and Higher IQ autism groups and the NT group.

### Preliminary Analyses

Preliminary analyses indicated that age was not significantly correlated with the SS, PS, or SJAS C-JARS scores in any of the groups (*rs* = − 0.16–0.15). Therefore, age was not included as a variable in the subsequent analyses. The Preliminary analyses also indicated that FSIQ was not significantly correlated with C-JARS scores in the Higher IQ autism and NT groups (*rs* = − 0.16–0.18). However, FSIQ was correlated with PS (*r* = 0.52, *p* < 0.001) and SJAS scores (*r* = 0.44, *p* < 0.007) but not SS scores (*r* = − 0.21, *p* = 0.23) in the Lower IQ autism group. More specifically, both PS and SJAS scores showed positive, moderate-to-strong correlations with Verbal IQ (*rs* = 0.67 and 0.57, respectively, *ps* < 0.001), but their correlations with Nonverbal IQ were not significant. When the Lower IQ and Higher IQ autism groups were combined, the PS and SJAS scores positively correlated with Verbal IQ (PS: *r* = 0.30, *p* < 0.001; SJAS: *r* = 0.26, *p* = 0.01). Since FSIQ was not a significant correlate of the dependent variables in two groups, IQ was not used as a covariate in the group effects analyses associated with the C-JARS scores.

### Diagnostic Group Differences on the SS, PS, and SJAS Scores

A 3 (Group) X 2 (Sex) MANOVA for the C-JARS SS and PS scores was conducted to examine how well parent report on the C-JARS distinguished children in the diagnostic groups. The results revealed a significant multivariate effect for group, *F* (4, 288) = 53.23, *p* < 0.001, Wilks’ Λ = 0.33, that was associated with a strong effect size, partial η^2^ = 0.43. Pairwise comparisons with a Bonferroni correction indicated that the NT group exhibited significantly lower SS scores but significantly higher PS scores than both autism IQ groups (see Table 3). The autism groups did not differ on the SS scores, but the Higher IQ autism group had a significantly higher PS score than the Lower IQ autism group (see Table 3).

**Table 3:** Group means and standard deviations for the C-JARS scores

Characteristics	Lower IQ Autism		Higher IQ Autism		NT	
	*n*	M (SD)	*n*	M (SD)	*n*	M (SD)
SS SCORES						
Female	7	2.15 (0.55)^c^	12	2.32 (0.43)^c^	22	0.76 (0.45)^a,b^
Male	29	2.23 (0.53)^c^	41	2.05 (0.72)^c^	40	0.74 (0.46)^a,b^
Total	36	2.22 (0.53)^c^	53	2.11 (0.55)^c^	62	0.75 (0.46)^a,b^
PS SCORES						
Female	7	1.63 (0.93)^c,b^	12	2.07 (0.81)^a,c^_m_	22	3.65 (0.31)^a,b^_m_
Male	29	1.96 (0.55)^c^	41	2.48 (0.72)^c^_f_	40	3.33 (0.50)^a,b^_f_
Total	36	1.90 (0.63)^c^	53	2.39 (0.75)^c^	62	3.44 (0.47)^a,b^
SJAS SCORES						
Female	7	− 0.53 (1.40)^c^	12	− 0.25 (1.06)^c^_m_	22	2.90 (0.56)^a,b^
Male	29	− 0.27 (0.88)^c^	41	0.43 (1.2)^c^_f_	40	2.58 (0.79)^a,b^
Total	36	− 0.32 (0.99)^c^	53	0.28 (1.20)^c^	62	2.69 (0.73)^a,b^

SS SCORES = C-JARS Social Symptom scale. PS SCORES = C-JARS Prosocial scale. SJAS = C-JARS Summary joint attention score. Autism = autism spectrum disorder. NT = neurotypical

_m_Within group gender differences with females significantly different from males, *p* < .05

_f_Within group gender differences with males significantly different from females, *p* < .05

^a^Significantly different from Lower IQ Autism group, *p* < .001

^b^Significantly different from Higher IQ Autism group, *p* < .01

^c^Significantly different from TD group, *p* < .001

A significant main effect for sex was not observed, *F* (2, 144) = 0.65, *p* = 0.52, Wilks' Λ = 0.99, partial η^2^ = 0.01. However, a Group X Sex interaction,* F* (4, 288) = 3.16, *p* = 0.02, Wilks' Λ = 0.92, was revealed with a weak effect size, partial η^2^ = 0.04 (see Table 3). Follow-up analyses revealed a significant group effect on the SS scores, *F* (2, 145) = 116.33,* p* < 0.001, partial η^2^ = 0.62, and the PS scores, *F* (2, 145) = 81.71, *p* < 0.001, η^2^ = 0.53. Follow-up analyses of the Group X Sex interaction revealed a significant interaction for PS scores, *F* (2, 144) = 5.00,* p* = 0.01, partial η^2^ = 0.07, but not for SS scores, *F* (2, 144) = 1.04, *p* = 0.36, partial η^2^ = 0.01 (see Table 3). Bonferroni-adjusted pairwise comparisons indicated there was no difference between males and females on the PS scores in the Lower IQ autism group. However, females in the Higher IQ autism group displayed significantly lower PS scores than did males in this group, *p* = 0.04 (Table 3). Alternatively, the opposite pattern was observed in the NT group, where parents reported higher PS scores for females than males, *p* = 0.04 (Table 3).

Univariate analyses also revealed a comparable strong main effect for group on the C-JARS SJAS score, *F* (2,135) = 41.60, *p* = 0.02, partial η^2^ = 0.98 (see Table 3), and a weak but significant Group X Sex interaction effect, *F* (2,135) = 3.06, *p* = 0.05, partial η^2^ = 0.04 (See Table 3). Here post-hoc analyses indicated that parents reported significantly lower SJAS for females than males in the Higher IQ autism group, *p* = 0.03, but not in the Lower IQ autism group or in the NT group (see Table 3). The mean SJAS score in Higher IQ autism females was negative because, as a group, they had lower PS scores than SS scores (Table 3). However, post-hoc paired t-tests indicated that the difference in their PS and SS scores are not significant. On the other hand, post-hoc analyses indicated that males in the Higher IQ autism group tended to receive significantly higher PS scores than SS scores, *t*(40) = 2.30, *p* = 0.027, *d* = 0.36 (Table 3).

### Diagnostic Group Discriminant Validity

Previous research indicated that preschool measures of joint attention can identify children with autism versus controls with significant sensitivity and specificity (0.82–0.92, Dawson et al., 2004; Mundy et al., 1986). A two group (autism vs. NT) discriminant function analysis was computed in this study to determine if the school-aged measures of the C-JARS displayed comparable sensitivity and specificity. Analysis of the SJAS scores indicated that lower scores were a significant identifier of the autistic and non-autistic participants in this study, Wilks’ Λ = 0.36, χ^2^(1) = 149.50, *p* < 0.001, sensitivity = 86.52%, specificity = 93.5%. The ROC analysis for the SJAS score indicated that a cutoff score of 1.59 identified 96% of positive cases of autism under the ROC curve. For comparison, with another measure in this study a discriminant function analysis of the Vineland-II Socialization scores resulted in a sensitivity estimate of 93.4% and specificity 87.8%, Wilks’ Λ = 0.40, χ^2^(1) = 144.50, *p* < 0.001.

Analyses also indicated that higher scores on the SS scale correctly identified 86.5% of the autism group and 93.5% of the NT group, Wilks’ Λ = 0.35, χ^2^(1) = 158.81, *p* < 0.001. The ROC analysis indicated that scores higher than 1.32 on the SS scale constituted a cutoff indicator of 97% of positive cases under the ROC curve in this sample. Analyses of the PS scale indicated that lower scores were a significant group identifier with sensitivity = 82%, specificity = 93.5%, Wilks’ Λ = 0.52, χ^2^(1) = 97.36, *p* < 0.001. The ROC analysis indicated that a cutoff score of 2.72 on the PS scale identified 91% of autism cases under the ROC curve.

### Construct Validity

The construct validity of the C-JARS was evaluated by examining the hypothesis that the C-JARS scores would be significantly correlated with the TMCQ Affiliation scale scores, ADOS-2 SA scores and Vineland-II Socialization scores. Conversely, the C-JARS scores were not expected to be correlated with the language (CELF-5) and anxiety (MASC-2) scores. The group means and comparative statistical data for those measures are provided in Table 4. As expected, the diagnostic groups differed on CELF-5 RLI and ELI scores and on all the Vineland-II scores (see Table 4). Two other notable observations were that parents reported higher MASC-2 Generalized Anxiety Disorder (GAD) scores for the Higher IQ autism group than the two other groups and, reciprocally, lower TMCQ Surgency scores compared to the other groups (Table 4).

**Table 4:** Group means and standard deviations for measures of language, anxiety, adaptive behavior, and temperament

Characteristics	Lower IQ Autism		Higher IQ Autism		NT	
	*n*	Mean (SD)	*n*	Mean (SD)	*n*	Mean (SD)
CELF-5						
RLI	12	56.50 (6.29)^b,c^	49	91.69 (19.77)^a,c^	57	109.68 (13.22)^a,b^
ELI	12	53.67 (6.05)^b,c^	49	89.67 (23.81)^a,c^	57	113.93 (12.66)^a,b^
MASC-2 GAD	27	51.78 (13.46)^b^	35	59.46 (12.15)^a,c^	47	46.49 (8.29)^b^
Vineland-II						
Receptive Language	35	8.49 (3.6)^b,c^	53	11.77 (2.9)^a,c^	61	15.90 (1.6)^a,b^
Expressive Language	35	7.37 (2.9)^b,c^	53	12.10 (2.8)^a,c^	61	16.00 (2.2)^a,b^
Socialization	35	57.11 (14.70)^b,c^	53	77.08 (17.84)^a,c^	61	112.20 (14.9)^a,b^
TMCQ						
Surgency	35	3.37 (0.38)^b^	52	3.08 (0.50)^a,c^	62	3.32 (0.40)^b^
Negative Affect	35	2.70 (0.59)^c^	52	2.87 (0.55)^c^	62	2.13 (0.48)^a,b^
Effortful Control	35	2.89 (0.43)^c^	52	2.93 (0.39)^c^	62	3.42 (0.43)^a,b^
Affiliation	35	3.26 (0.67)^c^	52	3.51 (0.63)^c^	62	4.05 (0.55)^a,b^

Scoring Metrics: CELF-5 Standard Scores, Mean = 100, SD = 15; MASC-2 T-Scores, Mean = 50, SD = 10; Vineland-II Receptive and Expressive Language v-scores, Mean = 15 and SD = 3; Vineland-II Socialization Standard Scores, Mean = 100, SD = 15; TMCQ, Mean scale scores with range 1 to 5

^a^Significantly different from Lower IQ Autism group, *p* < .01

^b^Significantly different from Higher IQ Autism group, *p* < .01,

^c^Significantly different from TD group, *p* < .01

*CELF*-5 Clinical Evaluation of Language Fundamentals, Fifth Edition. *RLI* Receptive Language Index. *ELI* Expressive Language Index. *MASC*-2 Multidimensional Anxiety Scale for Children, Second Edition. *Vineland*-II Vineland Adaptive Behavior Scales, Second Edition. *TMCQ* Temperament in Middle Childhood Questionnaire. Autism = autism spectrum disorder. *NT* neurotypical

### Convergent Validity Data

Correlations pertinent to the primary hypothesis about the construct validity of the C-JARS are presented in Table 5. The validity of the C-JARS scores was supported by evidence of significant positive correlations between parent report on the TMCQ Affiliation scale and the C-JARS PS and SJAS scores in all three groups; Lower IQ autism: *r*s = 0.56 and 0.43, respectively; Higher IQ autism: *rs* = 0.52 and 0.51, respectively; and NT: *rs* = 0.49 and 0.45, respectively (*p* < 0.01 for all correlations). Alternatively, the SS scale was negatively correlated with TMCQ Affiliation scores in all groups, but this association was only significant in the Higher IQ autism group (*r* = − 0.40, *p* < 0.01, see Table 5).

**Table 5:** Correlation construct validity data for the CJARS

	Lower IQ Autism			Higher IQ Autism			NT		
	SS	PS	SJAS	SS	PS	SJAS	SS	PS	SJAS
ADOS-2									
SA Total	0.24	− 0.45**	− 0.42	0.45**	− 0.33	− 0.41**	–	–	–
RRB Total	0.02	− 0.20	− 0.14	0.32	− 0.30	− 0.34	–	–	–
CELF-5									
RLI	− 0.12	0.43	0.31	− 0.04	− 0.02	0.01	0.05	0.13	0.05
ELI	− 0.39	0.32	0.40	− 0.05	0.03	0.04	− 0.07	0.08	0.10
Vineland-II									
Recep Lang	− 0.36	0.51**	0.52**	− 0.51**	0.54**	0.57**	− 0.07	0.15	0.14
Express Lang	− 0.32	0.69**	0.61**	− 0.54**	0.63**	0.65**	− 0.11	0.24	0.22
Socialization	− 0.39	0.65**	0.62**	− 0.54**	0.67**	0.67**	− 0.25	0.30	0.35**
TMCQ									
Affiliation	− 0.14	0.56**	0.43**	− 0.40**	0.52**	0.51**	− 0.21	0.49**	0.45**

*SS* C-JARS Social Symptom Scale, *PS* C-JARS Prosocial Scale, *SJAS* C-JARS Summary Joint Attention Scale, *SA* Social Affect score on the ADOS-2. *RRB* Restricted and Repetitive Behavior scores on the ADOS-2. *CELF*-5 Clinical Evaluation of Language Fundamentals, Fifth Edition, *RLI* Receptive Language Index and *ELI* Expressive Language Index. *TMCQ* Temperament in Middle Childhood Questionnaire

^**^p < .01

Convergent validity support for the C-JARS was also provided by the observations of negative correlations between the ADOS-2 SA scores and the C-JARS PS (*r* = − 0.45, *p* < 0.01) and SJAS (*r* = − 0.42, *p* < 0.01) scores in the Lower autism IQ group. In the Higher IQ autism group the SJAS scores were also negatively correlated with the ADOS-2 SA scale (*r* = 0.41, *p* < 0.01), but the correlation with PS scores were not significant in this study (*r* = 0.33,* p* > 0.05). However, ADOS-2 SA scores were significant and positively correlated with the C-JARS SS scores in the Higher IQ autism group (*r* = 0.45, *p* < 0.01), this was not the case for the Lower IQ autism group (see Table 5).

Additional support for the convergent validity of the C-JARS was provided by data from the Vineland-II Socialization scale. The Socialization scale scores were positively associated with the PS (*r* = 0.65,* p* < 0.001) and SJAS scores (*r* = 0.62, *p* < 0.001) in the Lower IQ autism group. In the Higher IQ autism group the C-JARS PS and SJAS scores were also positively correlated with Vineland-II Socialization scores (*rs* = 0.67, and 0.67, respectively, *ps* < 0.01). Furthermore, the Socialization scale was negatively correlated with the C-JARS SS scores in this group (*r* = − 0.54, *p* < 0.01). In the NT group the C-JARS SJAS scores were only significantly associated with Vineland-II Socialization scores (*r* = 0.35, *p* < 0.05).

### Divergent Validity Data

Support for the divergent validity of the C-JARS was provided by the observation that none of the C-JARS scores significantly correlated with the ADOS-2 RRB Total scores in the Lower IQ Autism group (*rs* = 0.20-0.02) and Higher IQ Autism group (*rs* = − 0.34–0.32), consistent with previous observations (Mundy et al., 2017). It was also the case that ADOS-2 SA Total and RRB Total scores were moderately correlated in both the Higher IQ autism group (*r* = 0.43, *p* = 0.001) and the Lower IQ autism group (*r* = 0.50, *p* < 0.01). This is consistent with observations from other studies (de Bildt et al., 2009; Hus et al., 2014).

Data from the Vineland-II Scales also raised a challenge to the divergent validity of the C-JARS. Counter to expectations, the C-JARS scores were significantly correlated with the expressive and receptive language scores from the Vineland-II Communication subscale. Positive associations for PS and SJAS scores with expressive language were observed in both autism groups (see Table 5). Significant language correlations with the SS scale were also observed for the Higher IQ autism group but not the Lower IQ group (Table 5). However, the Vineland-II language scales were not correlated with the C-JARS scores in the NT group (Table 5). These data raised the possibility that the C-JARS scores were not independent of parent perception of their children’s language use in the autism samples. This may have mediated the observed associations between C-JARS scores and parent report on the TMCQ Affiliation and Vineland-II Socialization scores. However, after controlling for parent report on the Vineland-II receptive language scores, parent report of TMCQ Affiliation remained significantly associated with the PS scores for the Lower IQ autism group (partial *r* = 0.43, *p* < 0.013), and with the CJARS PS and SJAS scores in the Higher IQ autism group (partial *r* = 0.48 and 0.47, respectively, *ps* < 0.001). The C-JARS PS also remained significantly associated with Vineland-II Socialization scores after controlling for expressive language scores in the Lower IQ autism group (partial *r* = 0.46, *p* = 0.007) and with the C-JARS PS and SJAS scores in the Higher IQ autism group (partial *r* = 0.45, *p* < 0.001 and partial *r* = 0.44, *p* < 0.001, respectively).

It was also the case that none of the associations between the CELF-5 and C-JARS scores were significant for the Higher IQ autism and NT groups (*rs* = − 0.07–0.13, *ps* > 0.35; Table 5). Only a subset of 14 children in the Lower IQ autism group received scores on the CELF RLI and 12 received scores on the ELI. Nevertheless, the correlations of CELF-5 and the C-JARS scores in this subsample of the Lower IQ autism group were not significant (*rs* = − 0.39–0.43, *ps* > 0.13).

Support for divergent validity was also challenged by the observation of an unanticipated pattern of correlations between parent report MASC-2 anxiety measure and the C-JARS scores across the groups. MASC-2 GAD ratings were positively associated with PS scores in the Lower IQ autism group (*r* = 0.48, *p* < 0.01). Examination of the MASC-2 Social Anxiety, Physical Symptoms, Harm to Self, and Panic Disorder sub-scale scores indicated the PS scores were only significantly associated with Harm to Self (*r* = 0.71, *p* < 0.01) in the Lower IQ autism group. This unexpectedly suggested that children with higher perceived anxiety about harm-to-self displayed more joint attention and sharing of experience with parents in this group.

Alternatively, as might be expected, MASC-2 GAD scores were negatively correlated with PS (*r* = -0.40) and SJAS (*r* = -0.40) scores in the Higher IQ autism group, although these were not significant in this study (*p* < 0.02). Nevertheless, this suggested that higher levels of anxiety may be associated with lower joint attention and sharing experience behaviors in this subsample. Interestingly, the difference between the correlations of MASC-2 GAD with PS scores across the two autism groups was significantly different, *F* (1,64) = 11.46, *p* = 0.00 l. Moreover, a pattern of correlations was observed for the NT groups that was similar to the Higher IQ autism group. NT group MASC-2 GAD scores were negatively related to SJAS scores (*r* = − 0.33, *p* = 0.03) and, unique to this group, anxiety was also significantly positively correlated with their SS scores (*r* = 0.46, *p* < 0.01). This raise the possibility child anxiety may be associated with difference in parent report on the C-JARS PS scores and that this effect may be conditional on the IQ in the autistic children.

### C-JARS Scores and Effortful Control, Negative Affect

Other notable correlation data indicated that TMCQ Effortful Control was negatively associated with C-JARS SS scores (*r* = − 0.46, *p* < 0.01) but positively associated with PS (*r* = 0.41, *p* < 0.01) and SJAS (*r* = 0.47, *p* < 0.01) scores in the Higher IQ autism group. These associations were largely due to correlations between TMCQ Inhibitory Control and the SS (*r* = − 0.46, *p* < 0.001), PS (*r* = 0.46, *p* < 0.001), & SJAS (*r* = 0.50, *p* < 0.001) scores in the Higher IQ autism group. Inhibitory control was also correlated with PS scores in the NT group (*r* = 0.42 *p* < 0.001). TMCQ Effortful Control and Inhibitory Control were not significantly correlated with C-JARS scores in the Lower IQ autism group.

Parent report on the TMCQ Negative Affect scale was also related to the SS (*r* = 0.50, *p* < 0.01), PS (*r* = − 0.44, *p* < 0.01), and SJAS (*r* = − 0.51, *p* < 0.01) scores in the Higher IQ autism group and the SS (*r* = 0.32, *p* = 0.01) scores in the NT group. Again, however, there were no significant C-JARS associations with Negative Affect observed for the Lower IQ autism group. Alternatively, there was no evidence that TMCQ Surgency was related to C-JARS scores in any of the groups (*rs* = − 0.16–0.23).

## Discussion

Symptoms of atypical development of joint attention and sharing of experience are characteristic of the preschool development of autism (e.g., Franchini et al., 2019). Recent studies using objective methods also suggest that joint attention impairments remain an abiding characteristic of the phenotype of autism in childhood and adolescence (e.g., Baranek et al., 2013; Bauminger et al., 2008; Colombi et al., 2009; Dykstra Steinbrener & Watson, 2015; Mundy et al., 2016; Nowell et al., 2018; Oberwelland et al., 2017; Redcay et al., 2013; Reddy et al., 2002). The patterns of data reported in this study were consistent with findings of the latter studies and indicate parent observations on the C-JARS provide a valid means of measuring differences in the development of joint attention, the spontaneous sharing of experiences, and participation in joint action activities in childhood development in autistic children.

In terms of psychometric data, and consistent with previous data (Mundy et al., 2017), the factor analytics data indicated the C-JARS items largely reflect a single common factor. That is, it is a factor held in common by items that variously assess joint attention, spontaneously sharing experiences, and engaging in cooperative behavior. Both the SS and PS scales of the C-JARS reflect this common factor and both scales displayed an acceptable level of internal consistency.

Group difference data supported the validity of the C-JARS as an instrument that is sensitive to potentially important differences in the school aged development of autistic children. Parent report on the C-JARS indicated that Lower IQ and Higher IQ autism groups displayed significantly higher Symptom Scale (SS) scores but lower Prosocial Scale (PS) and Summary Joint Attention Scale (SJAS) scores than their neurotypical peers. Moreover, large effect sizes were observed for the group differences on the C-JARS in this study such that, for example, the SJAS scores identified 86.5% of the 89 autistic children and 93.5% of 62 NT children. These data were comparable to the degree of autism vs. typical group separation that has been observed for preschool measures of joint attention in studies by Dawson and colleagues (2004) and Mundy and colleagues (1986). Other studies have indicated that measures of joint attention/spontaneous sharing of experience and collaborative/cooperative behaviors provide valid information about the social symptoms and the prosocial development of autistic children (Nowell et al., 2018). However, this study provides some of the first evidence of the significant diagnostic discriminative power that may characterize such measures.

It was also the case that evidence of the discriminant validity of the C-JARS was equal to that of the Vineland-II Socialization scores in this sample. The latter is considered to be a fundamental measure of aspects of the social phenotype of autism in childhood (Klin et al., 2007; Kraijer, 2000). However, the added value of the C-JARS stems in part from its theory-based focus on a specific aspect of the social symptoms of autism. Research suggests that early intervention may have a positive impact on this symptom dimension leading to improved outcomes (Kasari et al., 2008; Murza et al., 2016). Nevertheless, the data in this study and others suggest that measurable differences in joint attention and spontaneous sharing of experience persist into childhood for many autistic children. Improved measurement may be useful for childhood outcome studies of preschool intervention effects. Equally important, improved measurement may be a necessary step for the development and evaluation of targeted *childhood* joint attention intervention methods that could lead to continued improvement in this domain after preschool and through adolescence. Indeed, there is a general need to develop outcome measures that are sensitive to change in the social symptoms of autism (Anagnostou et al., 2015; Mazurek et al., 2020). To that end, the Likert scaling of C-JARS items was chosen to enable the SS, and especially the PS scale to contribute to filling the need for childhood social outcome measures in autism research. While the data in this study do not directly address the sensitivity of the C-JARS scores to change in childhood, the collection of such data would be an informative next step.

The data in this study also provided convergent evidence of the construct validity of the C-JARS. In this regard the observation that the C-JARS ratings were significantly correlated with parent ratings on the TMCQ Affiliation scale were especially important because theory and research has previously indicated that joint attention and the social sharing of experience is associated with the development of a positive sense of relatedness, affiliation, and friendship with other people (Freeman et al., 2015; Mundy & Sigman, 2006; Wolf & Tomasello, 2020; Wolf et al., 2016). It was also noteworthy that parent report of Surgency on the TMCQ was not correlated with C-JARS in any group, suggesting that correlations with the Affiliation scale were independent of parent perceptions of differences in the general level of extraversion displayed by their children.

Understanding the nature of the development of affiliative behavior is important to autism science (Vivanti et al., 2016). It is also important to the hypothesis that metabolic processes specifically involved in the regulation of affiliative behavior development may contribute to the complex etiology of autism, (Insel, 2010; Yrigollen et al., 2008) as well as to joint attention development (Gangi et al., 2014; Hopkins et al., 2014; Stavropulos & Carver, 2013; Wade et al., 2014. It may well be that parent report on a narrow-band assessment of joint attention, such as the C-JARS, could contribute novel and more specific information about the association between genes and the development of affiliative behavior and social symptoms in autism.

Additional data on construct validity indicated that the C-JARS PS and SJAS scores were significantly negatively correlated with ADOS-2 SA scores in both the Lower IQ and Higher IQ autism groups. Equally important was the observation that all C-JARS scores (SS, PS and SJAS) were significantly associated with the range of individual differences in general social development on the Vineland-II Socialization subscale in both autism groups, as well as NT group. Moreover, the CELF-5 standardized language development scores were not correlated with and the C-JARS. Thus, individual differences in language did not appear to be associated with parent report on the C-JARS in the autism groups. The observation that the correlation between C-JARS scores and the Vineland-II Socialization scores could not be explained by individual differences in parent report of their children’s expressive language on the Vineland-II provide additional support for this conclusion.

All of the foregoing suggests that the C-JARS SS and PS scales, as well as the SJAS score may be valid measures of childhood social outcomes in developmental and clinical research for autism. Anagnostou and colleagues (2015) reported that they could identify only six valid and reliable childhood social outcome measures available for autism research. Only two of these included measures of joint attention, but those were limited to outcomes for 2- to 6-year-olds. The C-JARS extends the age range of this type of outcome measure for research in childhood and adolescent development. Moreover, the C-JARS may be sensitive to heretofore unrecognized characteristics of the social development of autistic females. Parents reported that females in the Higher IQ autism group displayed lower average PS scores than SS scores. This pattern of observations was significantly different from the pattern displayed by males in this group, as well as males and females in the NT group. However, this interaction was associated with a weak effect size and the sample size of females in this study was modest. Therefore, it warrants a cautious interpretation. Nevertheless, the characterization of autism in females is a priority for future research (Estrin et al., 2021; Wood-Downie et al., 2021) and these data are notable because they raise the possibility that the C-JARS may contribute to the study of sex differences in childhood social development in autism.

Additional information about the convergent and discriminant construct validity were provided by several other observations. The TMCQ Effortful Control scores were correlated with all the C-JARS scores in the Higher IQ autism group and with PS scores in the NT group. Parent report of effortful control have been observed to relate to objective measures of the executive control of attention (Simmonds et al., 2007). Therefore, the data indicate that the executive control of attention and self-regulation contribute to differences in joint attention development in the development of autism (Jahromi et al., 2019; Van Hecke et al., 2012). However, Effortful Control was not associated with C-JARS scores in the Lower IQ autism group. It was unclear why this group difference in the associations of C-JARS with Effortful Control were observed. However, the data in this study also indicated that children’s performance on a standardized IQ measure was related to C-JARS scores in the Lower IQ autism group but not the Higher IQ autism group. These observations raise the possibility that variance in child cognitive factors may impact parent report on the C-JARS across Higher and Lower IQ groups of autistic children.

The C-JARS was designed for assessment of children without intellectual disability (Mundy et al., 2017) and these data suggest that more data will be required to understand its validity with children with intellectual disability. Similarly, a varied pattern of correlations suggested that there may be important differences between the impact of child anxiety on parent reports of C-JARS social behaviors in autistic children with and without intellectual disability. Parent reports of anxiety, and specifically MASC-2 Harm Avoidance, were positively correlated with the PS scale in the Lower IQ autism group. This may indicate harm avoidance motivates the spontaneous sharing experience with caregivers among Lower IQ autistic children. However, to our knowledge, this is a novel observation so it must be interpreted with caution. On the other hand, a significantly negative association between anxiety and the PS scores were observed in the Higher IQ autism group. This was consistent with studies showing parent reports of negative affect are correlated with reports of anxiety in autistic children (Ambrose et al., 2021; Usher et al., 2015). Finally, a significant association between the SS scores and anxiety were observed in the NT group. This set of observations suggests that emotional factors may impact C-JARS scores in different ways across different groups of children. More research will be needed to understand the impact of anxiety and other emotions on the nature and validity of the C-JARS for research on autism and childhood social development. However, these observations also suggest the C-JARS may provide a useful social outcome measure in the study of affective distress in children with autism.

## Summary and Limitations

The data in this study attest to the validity of the C-JARS and its promise to provide both a symptom index and positive social development index of joint attention and the spontaneous sharing of experience in the childhood development of autism. Nevertheless, the sampling method and sample size prevents the generalization of the data, especially the sensitivity, specificity, and ROC data, to population level estimates or clinical applications. Additional research with larger samples will be needed to address diversity, equity, and inclusion (DEI) related questions about the degree to which differences in the socioeconomic status, race/ancestry, culture, ethnicity, or other family factors intersect with C-JARS validity or its utility in autism research. Second, larger scale studies as well as developmental studies will be required to more definitively address the factor structure of the scale, the standardization of scale scores, and the sensitivity of its scores to development change and treatment outcomes.

Another limitation of this study is that the study relies singularly on parent report data and this common source of information may have impacted (inflated) the correlation data used to appraise the construct validity of the C-JARS. In addition, the parent report temperament measure (TMCQ) was primarily designed for studies with 7 to 10-year-old children, which also may have impacted the data reported in this study. Additional studies which combine parent report on the C-JARS with more data from independent behavioral observation will be needed to more comprehensively appraise the construct validity of the C-JARS. For example, the data in this study does not provide evidence that C-JARS parent report data is related to the types of joint attention behaviors most often studied in autism research. Hence, this study does not speak to a critical test of the validity of the C-JARS. This will require the examination of the associations of the C-JARS with objective measures of the development of joint attention in childhood, such as the *Attention-Following and Initiating Joint Attention Protocol* (Nowell et al., 2018). Alternatively, another approach to testing this assumption is suggested by the observation that clinical observations on several items of the ADOS-2 SA may be aggregated to constitute a joint attention factor scale (see Gotham et al., 2007, 2008; Harrison et al., 2016; Zachor & Ben-Itzchak, 2020). Data on the degree to which the C-JARS is specifically related to the “joint attention” items of the ADOS-2 SA scale could provide direct support, or refutation of the assumption that the C-JARS measures childhood behaviors that are part of the developmental continuous expression of the preschool joint attention symptom dimension of autism.
